# Fascia‐Level Temperature Kinetics During Multi‐Wavelength Diode Laser Irradiation: A Cadaveric Study

**DOI:** 10.1111/jocd.70905

**Published:** 2026-05-16

**Authors:** Kyu‐Ho Yi, Yerin Park, Hugues Cartier, Sebastien Garson, Benjamin Ascher

**Affiliations:** ^1^ You and I Clinic Seoul Republic of Korea; ^2^ Medical Research Seoul Republic of Korea; ^3^ Centre Médical Saint Jean Arras France; ^4^ Cabinet Médical Senlis France; ^5^ SIBUS Paris France

**Keywords:** cadaveric study, fascia‐level temperature kinetics, multi‐wavelength diode laser, noninvasive body contouring, subcutaneous fat thickness

## Abstract

**Background:**

Thermal energy‐based technologies are widely used for noninvasive body contouring; however, quantitative characterization of fascia‐level heat propagation in body tissues during multi‐wavelength diode laser irradiation remains limited.

**Objective:**

To assess fascia‐level temperature kinetics and regional differences in deep‐plane heating during stacking delivery of a multi‐wavelength diode laser (Fortra, Classys Inc., Korea) in human cadaver tissues.

**Methods:**

Three fresh‐frozen human cadavers underwent laser irradiation at the abdomen, anterior thigh, and lateral upper arm. Subcutaneous fat thickness was measured by ultrasonography. A single thermocouple was inserted to the muscle fascial plane to record fascia‐level temperature over time, and infrared thermography of the exposed measurement field was performed after tissue exposure to identify the local thermographic peak. Deep fascial arrival time, peak fascial temperature, exposed‐field thermographic peak temperature, and fascia‐level cumulative equivalent minutes at 43°C (CEM43) were evaluated. Regional comparisons were descriptive, with exploratory nonparametric testing.

**Results:**

Heat reached the muscle fascia fastest in the anterior thigh (4.9 ± 0.4 s), followed by the lateral upper arm (5.3 ± 0.5 s), and slowest in the abdomen (6.6 ± 0.6 s). These values represent mean ± SD of cadaver‐level means. Regional differences in fascial arrival time were exploratory but reached significance (Friedman test, *p* = 0.049). Exposed‐field thermographic peak temperatures were highest in the anterior thigh (70°C–72°C), followed by the lateral upper arm (68°C–71°C), and lowest in the abdomen (65°C–68°C).

**Conclusions:**

Under non‐perfused ex vivo conditions, fascia‐level thermal behavior during multi‐wavelength diode laser irradiation varied by anatomical region. Greater subcutaneous fat thickness was associated with slower fascial heat arrival, but did not fully explain regional variation. These findings are descriptive and do not establish volumetric heat distribution, histologic injury, or in vivo safety thresholds.

## 1 Introduction

Thermal energy‐based technologies have become central to noninvasive body contouring, providing alternatives to surgical fat reduction through controlled delivery of heat to cutaneous and subcutaneous tissues. Clinical applications using radiofrequency, laser, and high‐intensity focused ultrasound have demonstrated that appropriately dosed thermal exposure can induce collagen contraction, delayed tissue remodeling, and, under certain conditions, structural modification of subcutaneous adipose tissue [1, 2, 3, 4]. However, although these clinical outcomes imply biologically meaningful heat delivery, they do not directly define how thermal energy propagates over time to deeper anatomical planes within different body regions.

Among these modalities, diode laser systems offer distinct advantages related to wavelength‐dependent tissue interactions. Laser wavelength influences both the depth of energy deposition and the relative contribution of chromophore absorption, including melanin, water, and lipid components [5, 6, 7]. Representative published chromophore spectra support this wavelength dependence. For melanosomes, the absorption coefficient decreases with increasing wavelength and can be approximated by a power‐law relationship, yielding values of approximately 172 cm^−1^ at 755 nm, 137 cm^−1^ at 808 nm, 83 cm^−1^ at 940 nm, and 55 cm^−1^ at 1064 nm. In contrast, water absorption is relatively low at 755 and 808 nm (approximately 0.025 and 0.020 cm^−1^, respectively), increases markedly at 940 nm (0.267 cm^−1^), and remains higher at 1064 nm (approximately 0.12 cm^−1^). Published mammalian fat spectra likewise demonstrate a near‐infrared absorption band in the 930–980 nm region, supporting greater relative interaction of longer wavelengths with lipid‐rich subdermal tissues [7]. Accordingly, longer wavelengths in the near‐infrared range may permit a greater proportion of optical energy to reach subdermal and adipose compartments than shorter wavelengths, although the present study did not independently measure wavelength‐specific penetration depth.

Optimization of body contouring protocols, however, remains largely empirical. Unlike facial regions, where tissue thickness and anatomical boundaries are relatively consistent, body areas exhibit substantial regional variability in subcutaneous fat thickness, fibrous septal organization, and the depth to deep fascial planes [4, 8]. These anatomical differences limit the validity of inferring deep‐tissue thermal effects solely from surface‐level observations and suggest that identical energy delivery conditions may produce divergent subsurface thermal responses depending on the treatment site.

From a biophysical perspective, tissue heating during thermal energy treatments is governed by wavelength‐dependent optical absorption, thermal conduction, and subsequent heat diffusion across heterogeneous tissue layers. Experimental and simulation studies have shown that identical energy delivery conditions can result in different internal temperature profiles depending on tissue composition and depth distribution [7, 9, 10, 11]. Such effects are expected to be particularly relevant in body regions where subcutaneous fat thickness varies substantially across anatomical sites.

Despite the extensive clinical literature on noninvasive body contouring technologies, most prior investigations have emphasized post‐treatment clinical outcomes such as volumetric fat reduction, circumferential change, or safety assessment, rather than directly examining the kinetics of deep thermal propagation within body tissues [12, 13, 14, 15, 16]. Consequently, how delivered energy translates over time into measurable thermal exposure at deep tissue planes remains insufficiently characterized, particularly under controlled irradiation conditions. In this context, cadaveric fascia‐level measurement provides a practical experimental model because it permits direct temperature assessment at a reproducible deep anatomical reference plane while preserving regional differences in tissue thickness and architecture under standardized delivery conditions. Although such measurements do not reproduce perfusion‐corrected in vivo heat behavior and cannot define full volumetric temperature distributions, they can help characterize site‐dependent fascia‐level heat arrival in a controlled ex vivo setting.

Accordingly, the present cadaveric study was designed to assess fascia‐level temperature kinetics during multi‐wavelength diode laser delivery across different body regions. Using combined intratissue thermocouple measurement and infrared thermography of the exposed measurement field, this study aimed to characterize regional differences in fascial arrival time, peak fascial temperature, and thermographic peak temperature, and to explore how subcutaneous fat thickness relates to fascia‐level thermal response under these experimental conditions.

## 2 Methods

### 2.1 Study Design

This was a cadaveric study designed to quantify fascia‐level temperature kinetics in body tissues during fixed‐spot stacking delivery of a multi‐wavelength diode laser. Three human cadavers were included in the investigation. All specimens were fresh‐frozen cadavers and were thawed at room temperature for 24 h before experimentation. Before thermal assessment, each specimen was inspected for overt dehydration, surface disruption, prior surgical intervention, gross scarring, or dermatologic pathology that could materially affect heat conduction or tissue‐level temperature assessment.

Each cadaver underwent assessment in three predefined anatomical regions: the abdomen, anterior thigh, and lateral upper arm. Within each body region, a representative ROI was defined for thermal imaging analysis, and laser irradiation experiments were performed in triplicate at separate anatomically matched, non‐overlapping sites within the same region under identical preset conditions. Accordingly, a total of 27 experimental measurements were obtained for each assessment modality (3 cadavers × 3 anatomical regions × 3 repeated measurements).

Thermal assessment was performed using thermocouple measurements and infrared thermography. The muscle fascial plane was selected prospectively as a reproducible deep anatomical reference plane for regional assessment of time‐dependent heat propagation. No validated volumetric depth mapping was performed, and the study was not designed to establish a maximum penetration depth. Thermocouple measurements were used to determine heat arrival time to the fascial plane and to monitor real‐time temperature changes at this depth. Infrared thermography was performed only after tissue exposure and therefore provided a two‐dimensional temperature map of the exposed measurement field rather than intact skin‐surface temperature during treatment. For each repeated run, thermographic recording and thermocouple‐based temperature acquisition were performed at the same test site. Before irradiation, the cadavers were allowed to equilibrate in a climate‐controlled laboratory maintained at 22.0°C–23.0°C and 45%–55% relative humidity for at least 30 min. Baseline tissue temperature was recorded immediately before laser delivery; the mean pre‐irradiation surface temperature across all ROIs was 21.8°C ± 0.9°C, and the mean baseline deep‐plane temperature measured by thermocouple was 22.4°C ± 0.7°C. Prior to irradiation, subcutaneous fat thickness was quantified to establish baseline anatomical parameters relevant to subsequent thermal analysis.

To control for background temperature fluctuation and potential measurement artifact associated with probe placement, baseline thermocouple insertion control measurements were obtained at each test site before laser irradiation. After thermocouple placement, temperature was recorded for 30 s without laser activation to confirm thermal stability and absence of procedure‐related heating.

Human cadaver specimens were obtained through a legally authorized body donation program with prior donor consent for research and educational use and were handled with respect in accordance with institutional policies and applicable national regulations. All specimens and images were fully de‐identified.

### 2.2 Regions of Interest

The analysis included three body regions: the abdomen, anterior thigh, and lateral upper arm. For each region, a standardized square region of interest (ROI) measuring approximately 4 × 4 cm was marked on the skin surface. The abdominal ROI was positioned on the anterior abdominal wall approximately 3 cm lateral to the umbilicus. The anterior thigh ROI was centered at the midpoint between the anterior superior iliac spine and the superior border of the patella. The lateral upper arm ROI was defined along the lateral aspect of the upper arm between the deltoid insertion and the triceps muscle belly.

### 2.3 Device

Laser irradiation was conducted using a multi‐wavelength diode laser system (Fortra, Classys Inc., Seoul, South Korea). The system integrates four diode wavelengths (755, 808, 940, and 1064 nm) within a single handpiece and delivers them simultaneously during a single irradiation sequence. Under the experimental configuration used in this study, the programmed total composite fluence was evenly distributed across the four wavelengths, corresponding to fluence of 5 J/cm^2^ was applied. Independent spectroradiometric verification of wavelength‐specific output was not performed; therefore, wavelength‐specific contribution is described according to the programmed device setting.

**FIGURE 1:**
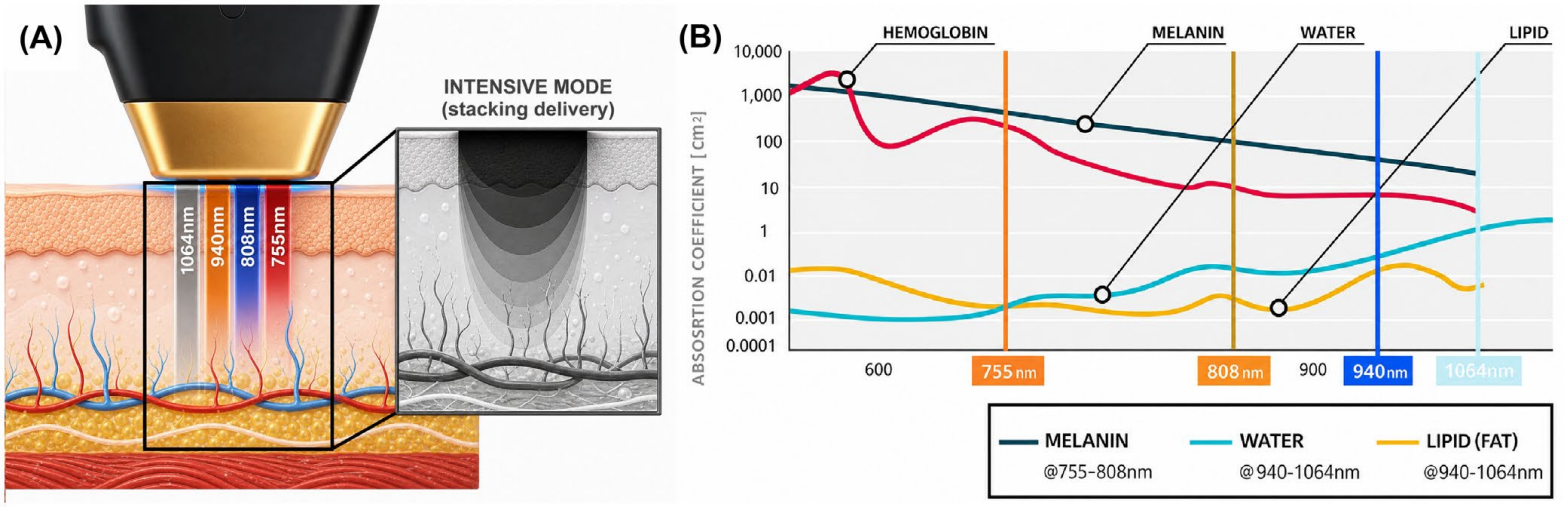
Multi‐wavelength diode laser irradiation and representative published optical absorption characteristics. (A) Simultaneous emission of four diode wavelengths (755, 808, 940, and 1064 nm) from a single handpiece during intensive stacking delivery. (B) Representative published optical absorption behavior of major cutaneous chromophores across the relevant wavelength range, including melanin, water, and lipid components.

The wavelength‐dependent behavior of the system is supported by representative published chromophore data [7]. At the melanosome level, absorption is higher at shorter wavelengths, with approximate absorption coefficients of 172 cm^−1^ at 755 nm and 137 cm^−1^ at 808 nm, decreasing to 83 cm^−1^ at 940 nm and 55 cm^−1^ at 1064 nm. In contrast, water absorption is low at 755 and 808 nm (approximately 0.025 and 0.020 cm^−1^, respectively), increases substantially at 940 nm (0.267 cm^−1^), and remains elevated at 1064 nm (approximately 0.12 cm^−1^). Published mammalian fat spectra also show a characteristic near‐infrared absorption band in the 930–980 nm region. Taken together, these constituent spectra support the hypothesis that the 755‐ and 808‐nm wavelengths are relatively more weighted toward superficial melanin‐associated absorption, whereas the 940‐ and 1064‐nm wavelengths may contribute relatively more to water‐ and lipid‐rich subdermal tissues (Figure 1).

The system incorporates a sapphire contact cooling interface with adjustable temperature settings of 0°C, −3°C, and −5°C. During irradiation, the cooling interface remains in continuous contact with the skin surface. Under these conditions, the sapphire tip acts as a superficial heat sink, limiting epidermal temperature elevation and altering the surface boundary condition for heat transfer. As a result, skin‐surface temperature during intact‐skin delivery is expected to remain lower than the temperature measured within deeper or exposed tissue planes; this should be considered when interpreting the thermographic peak values reported in the present ex vivo model.

In the present study, the INTENSIVE mode was used, in which multiple laser pulses were delivered repeatedly to the same location, resulting in rapid localized accumulation of thermal energy within a confined tissue volume. This mode was selected to evaluate fascia‐level heating under fixed‐site stacking conditions and not to demonstrate a validated mechanistic advantage over other delivery modes.

### 2.4 Delivery Parameters

All experiments were conducted using the INTENSIVE (stacking) mode to accumulate thermal energy at a fixed location within body tissues and to assess localized heat buildup under fixed‐site delivery conditions. Sapphire contact cooling was applied continuously at −3°C throughout irradiation to stabilize epidermal temperature. Laser parameters were standardized across all body regions. Irradiation was delivered at a total composite fluence of 5 J/cm^2^ per stack, representing the combined energy output of the four simultaneously emitted wavelengths (755, 808, 940, and 1064 nm). Thirty consecutive stacks were applied at a pulse repetition rate of 5 Hz, resulting in a total stacking duration of 6.0 s per region of interest and a cumulative composite fluence of 150 J/cm^2^ across the full sequence.

### 2.5 Fat Thickness Measurement

Baseline subcutaneous fat thickness was measured using ultrasonography at the center of the predefined region of interest prior to laser irradiation. Subcutaneous fat thickness was defined as the distance from the lower boundary of the dermis to the superficial fascia.

### 2.6 Thermal Measurements

Thermal measurements were performed using intratissue thermocouple recording and infrared thermography. Subsurface temperature was measured at a single deep‐plane location using a K‐type thermocouple with a diameter of 0.5 mm and a response time of less than 0.3 s. The thermocouple was inserted percutaneously and advanced perpendicular to the skin surface to the depth corresponding to the ultrasonographically measured distance from the skin surface to the muscle fascial plane at the ROI. Contact with the fascial plane was additionally identified by a consistent increase in insertion resistance. The same placement protocol was applied across repeated measurements, but the method does not provide continuous mapping across the dermis, fat, and intermediate tissue layers. Thermocouple recordings were used to assess temperature behavior at the muscle fascial plane during the 6‐s stacking period and the immediate post‐irradiation interval. Before experimentation, the thermocouple system was calibrated against a reference thermometer using a temperature‐controlled water bath over a range of 20°C–60°C. Calibration confirmed a measurement accuracy of within ±0.3°C. After probe placement, baseline temperature was recorded for 30 s before laser activation to confirm signal stability. Temperature signals were recorded at a sampling rate of 10 Hz, corresponding to a temporal resolution of 0.1 s. The primary thermocouple endpoints in this study were fascial arrival time, peak fascial temperature, and cumulative temperature behavior at the deep fascial plane during repeated 5‐Hz stacking delivery. At this depth, the recorded temperature profile primarily reflects conductive heat transfer and cumulative thermal buildup rather than ultrafast superficial thermal transients. Because the thermocouple response time was less than 0.3 s, 10‐Hz sampling was considered adequate to characterize the temporal profile of fascia‐level heating under the experimental conditions examined. Infrared thermography (FLIR, Wilsonville, OR, USA) was performed after tissue exposure following laser irradiation and was used to visualize heat distribution within the exposed field and identify the maximum thermographic temperature within that field.

### 2.7 Thermal Dose Calculation

To integrate the time–temperature history recorded at the deep fascial plane, thermal dose was additionally expressed as cumulative equivalent minutes at 43°C (CEM43), according to the Sapareto–Dewey formulation. CEM43 was calculated from the continuous thermocouple recordings using the equation:

$$\mathrm{CEM}43=\int_{0}^{t}R^{\left(43-T\left(t\right)\right)}dt$$

where *T(t)* is the measured fascial temperature at time *t*, *R* = 0.5 for *T* ≥ 43°C, and *R* = 0.25 for *T* < 43°C. Calculations were performed using the full temperature–time dataset obtained at a sampling rate of 10 Hz. For numerical implementation, the integral was discretized using the recorded temperature values.

### 2.8 Statistical Analysis

Triplicate measurements obtained within each cadaver and anatomical region were averaged to generate a single cadaver‐level value per region. Thus, summary values by region represent *n* = 3 cadaver‐level observations. Regional differences in fascial arrival time were evaluated using the Friedman test, treating cadaver as the matched block and region as the repeated condition. The association between peak fascial temperature and fascial arrival time was explored using Spearman rank correlation analysis across the nine cadaver‐region means. The relationship between subcutaneous fat thickness and fascial arrival time was explored using simple linear regression across the same cadaver‐region means. Because of the very small sample size, all inferential analyses were considered exploratory and were used to support descriptive interpretation rather than definitive statistical inference. A two‐sided *p* value of < 0.05 was considered nominally significant.

## 3 Results

### 3.1 Subcutaneous Fat Thickness

Subcutaneous fat thickness varied across cadaver specimens and body regions. At the abdominal site, measured subcutaneous fat thickness was 23.1 mm, 28.7 mm, and 30.4 mm across the three cadavers (mean 27.4 mm). In the anterior thigh, measured thickness values were 11.2 mm, 14.3 mm, and 17.1 mm (mean 14.2 mm). In the lateral upper arm, measured thickness values were 8.9 mm, 10.7 mm, and 13.7 mm (mean 11.1 mm). Because only three cadavers were included, these measurements are presented descriptively.

### 3.2 Deep Fascial Arrival Time by Body Region

**FIGURE 2:**
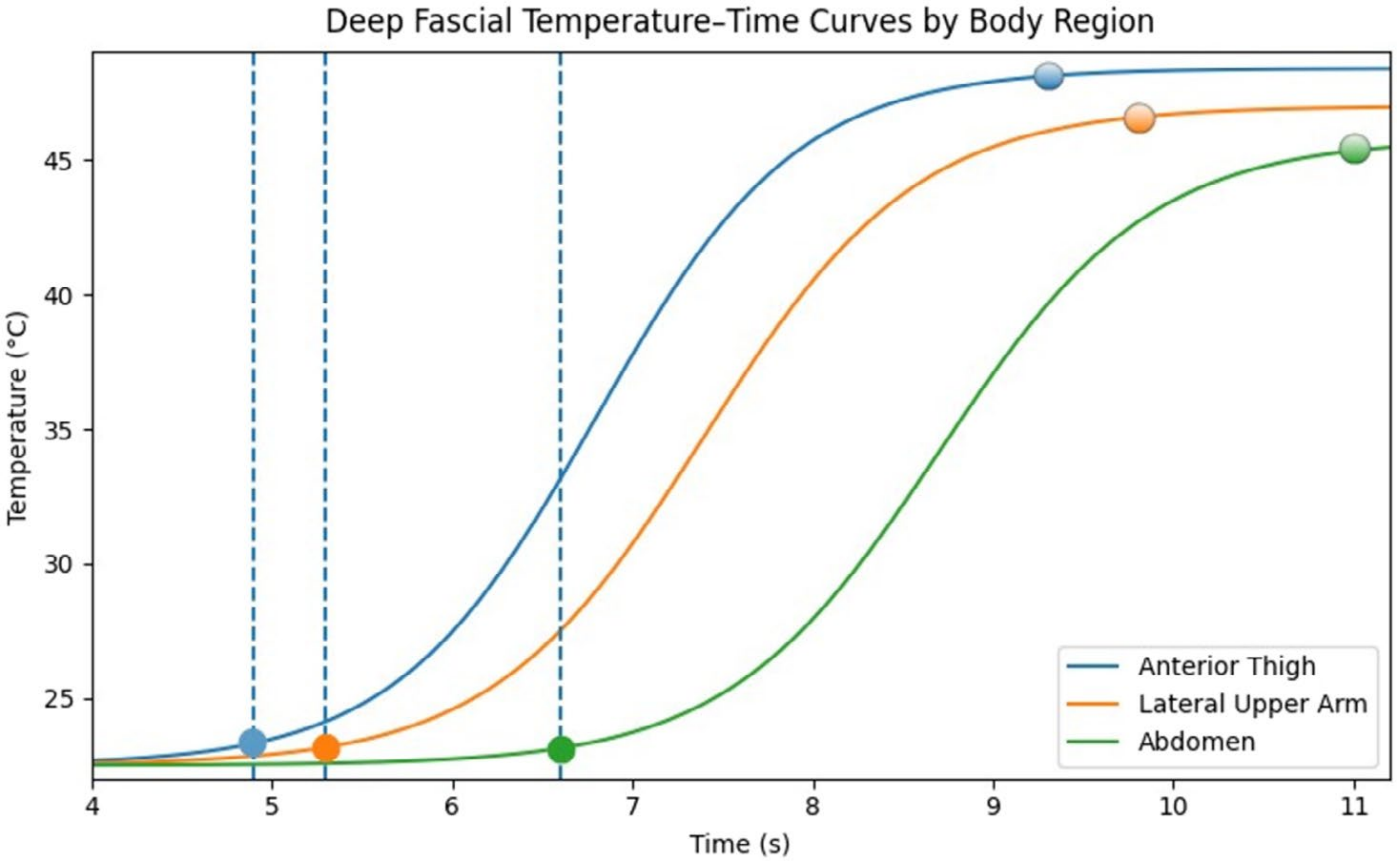
Deep fascial temperature–time curves by body region. Temperature–time curves are shown for the anterior thigh, lateral upper arm, and abdomen. The points at which each curve intersects the dashed vertical line indicate the reported deep fascial arrival time for that body region, whereas the circular markers at the right indicate the corresponding peak fascial temperatures.

Thermocouple measurements at the deep fascial plane demonstrated regional differences in time to fascia, peak fascial temperature, and cumulative thermal dose. Deep fascial arrival time was defined as the first time point at which the recorded temperature exceeded the baseline temperature by ≥ 0.5°C, indicating the onset of measurable heat transfer to the fascial plane. Values are presented as mean ± SD of cadaver‐level means (*n* = 3 cadavers per region). The anterior thigh showed the fastest deep fascial arrival, with a mean arrival time of 4.9 ± 0.4 s, followed by the lateral upper arm at 5.3 ± 0.5 s, whereas the abdomen demonstrated the longest arrival time, with a mean of 6.6 ± 0.6 s. Regional differences in fascial arrival time were evaluated exploratorily using the Friedman test based on cadaver‐level mean values across anatomical regions (*p* = 0.049). Peak fascial temperature was highest in the anterior thigh (48.4°C ± 0.9°C), followed by the lateral upper arm (47.0°C ± 0.8°C), and lowest in the abdomen (45.8°C ± 0.7°C). The deep fascial temperature–time curves by body region are shown in Figure 2. Consistent with these findings, fascia‐level CEM43 was highest in the anterior thigh (2.08 ± 0.35 min), followed by the lateral upper arm (1.54 ± 0.27 min), whereas the abdomen demonstrated the lowest cumulative thermal dose at the fascial plane (0.48 ± 0.19 min). Exploratory Spearman correlation analysis across cadaver‐region means showed an inverse association between peak fascial temperature and fascial arrival time (Spearman *ρ* = −0.965, *p* < 0.001). Exploratory linear regression analysis suggested that greater subcutaneous fat thickness was associated with delayed fascial arrival time (*β* = 0.103 s/mm, *R* ^2^ = 0.745, *p* = 0.003). These analyses should be interpreted cautiously because of the limited sample size, but they support the descriptive pattern that regions with faster heat arrival at the deep fascial plane tended to demonstrate higher peak fascial temperatures and greater cumulative thermal exposure during stacked diode laser delivery (Table 1).

**TABLE 1:** Fascial thermocouple‐based thermal parameters by body region.

Region	Peak fascial temperature (°C)	Time to fascia (s)	Fascia‐level CEM43 (min)
Abdomen	45.8 ± 0.7	6.6 ± 0.6	0.48 ± 0.19
Lateral upper arm	47.0 ± 0.8	5.3 ± 0.5	1.54 ± 0.27
Anterior thigh	48.4 ± 0.9	4.9 ± 0.4	2.08 ± 0.35

*Note:* Data are mean ± SD of cadaver‐level mean values (*n* = 3 cadavers per region).

### 3.3 Peak Temperature

**FIGURE 3:**
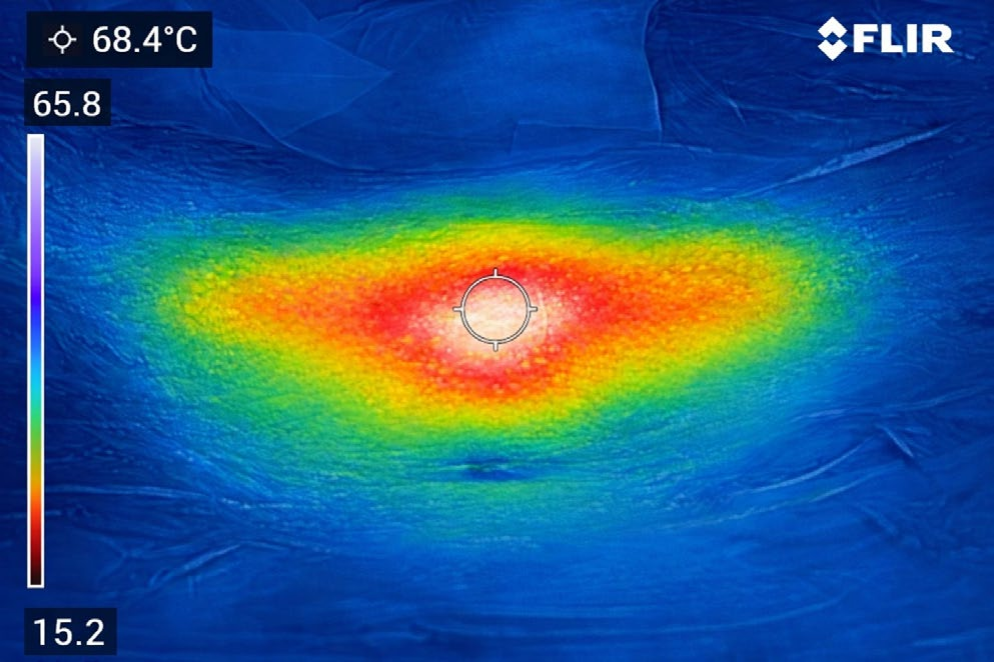
Infrared thermographic image of the abdominal region after tissue exposure following multi‐wavelength diode laser irradiation. Thermography obtained from the opened abdominal measurement field shows temperature elevation within the exposed subcutaneous plane after thermal delivery, with a peak temperature of 68.4°C at the center of the irradiation field. The image does not represent intact skin‐surface temperature during contact‐cooled treatment.

Infrared thermography was performed after completion of thermocouple measurements and after opening the measurement site to expose the subcutaneous fat layer and muscle fascial plane. Accordingly, the reported thermographic peaks reflect the exposed measurement field rather than intact skin‐surface temperature during contact‐cooled irradiation. Under identical irradiation parameters, the anterior thigh demonstrated the highest exposed‐field thermographic peak, reaching approximately 70°C–72°C, followed by the lateral upper arm at approximately 68°C–71°C. In contrast, the abdominal region exhibited lower thermographic peaks, generally in the range of 65°C–68°C, as demonstrated by infrared thermographic imaging (Figure 3).

## 4 Discussion

### 4.1 Deep‐Plane Thermal Propagation Across Body Regions

The present cadaveric study shows that heat reached the deep fascial plane at different rates depending on anatomical region, even when irradiation parameters and delivery technique were standardized. The anterior thigh showed the fastest fascial heat arrival, followed by the lateral upper arm, whereas the abdomen demonstrated the slowest fascia‐level heating. Overall, regions with thinner subcutaneous fat tended to show faster heat arrival at the deep fascial plane than the abdomen, which had the greatest adipose thickness and the longest arrival time.

These findings are consistent with prior experimental observations showing that adipose tissue can act as a thermal buffer because of its lower thermal conductivity relative to dermal and muscular tissues [2, 7]. Hyperthermia studies targeting adipocytes further suggest that effective deep thermal exposure depends on both energy magnitude and exposure duration [17]. In regions with thicker adipose compartments, a larger proportion of delivered energy may be retained or dissipated within superficial fat, thereby delaying transmission to deeper planes. In the present study, however, these considerations are offered only as biophysical context; the data do not directly establish a causal mechanism of vertical heat transfer.

**FIGURE 4:**
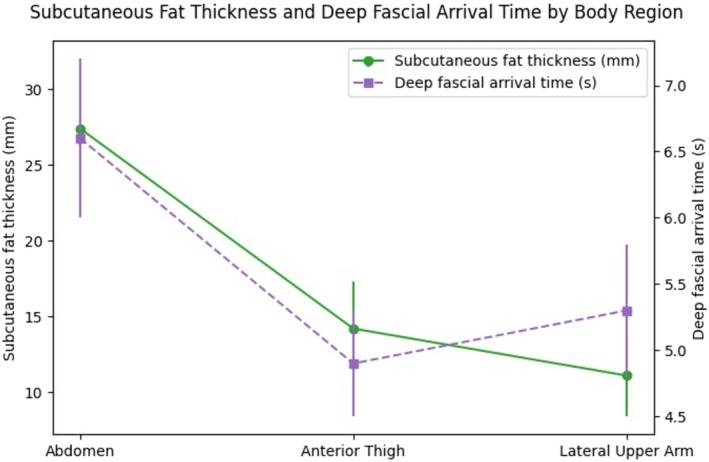
Dual‐axis plot showing subcutaneous fat thickness and deep fascial arrival time by body region. Mean subcutaneous fat thickness (mm) and deep fascial arrival time (s) are shown for the abdomen, anterior thigh, and lateral upper arm. Data points represent mean values.

However, the present results also indicate that the influence of subcutaneous fat thickness on fascia‐level heating is not absolute. Although thinner adipose layers generally favored faster fascial heat arrival, this relationship was not uniform across regions. Notably, the anterior thigh demonstrated faster deep fascial arrival than the lateral upper arm despite not having the thinnest subcutaneous fat layer (Figure 4). This pattern suggests that fascia‐level thermal response may also be influenced by additional structural factors, such as adipose compartment organization, fibrous septal architecture, and the relative contribution of underlying muscle at the treated site. These factors were not independently measured in the present study and are therefore best regarded as hypotheses for future investigation rather than established explanations.

In addition to time to fascia and peak fascial temperature, the present study incorporated fascia‐level CEM43 as a descriptive thermal dose metric derived from continuous thermocouple recordings at the deep fascial plane. This parameter complements point temperature measurements by integrating temperature magnitude and exposure duration into a single index of cumulative thermal burden. In the present dataset, anatomical regions with faster fascial heat arrival and higher peak fascial temperatures also demonstrated higher fascia‐level CEM43 values, with the anterior thigh showing the greatest cumulative thermal exposure, followed by the lateral upper arm and the abdomen. These observations remain descriptive because no histologic validation or injury modeling was performed.

Within this framework, fascia‐level thermal behavior should not be interpreted solely on the basis of time to fascia or peak fascial temperature, but rather as part of a broader thermal profile that also includes cumulative fascial heat exposure. This interpretation is consistent with fundamental thermal diffusion principles, whereby regional thermal behavior may be shaped by the combined influence of tissue composition, heat accumulation, and depth‐dependent conduction [7, 11]. Nevertheless, the present study measured temperature at only one deep plane and did not perform volumetric mapping; therefore, it cannot define the full spatial distribution of heat across intervening tissues.

### 4.2 Peak Thermal Exposure in the Exposed Measurement Field

Infrared thermography demonstrated regional variation in peak temperatures within the exposed measurement field. The anterior thigh showed the highest thermographic peaks (approximately 70°C–72°C), followed by the lateral upper arm (approximately 68°C–71°C); whereas the abdomen exhibited comparatively lower values (approximately 65°C–68°C). Because thermography was obtained after tissue exposure, these values represent local temperatures within the opened field rather than cooled epidermal surface temperatures during intact‐skin treatment. They therefore should not be interpreted as direct surface temperatures encountered clinically.

Prior experimental and clinical literature indicates that subcutaneous adipose tissue can respond differently across a range of thermal exposures, with moderate hyperthermia being associated in some settings with adipocyte injury and subsequent remodeling [18, 19, 20]. These studies provide useful context for interpreting the magnitude of heating observed in the present work. However, the current cadaveric study did not include histology, tissue viability assessment, or in vivo follow‐up and therefore cannot determine whether the recorded temperature profiles would correspond to selective adipocyte injury, reversible hyperthermia, or overt thermal damage in living tissue.

Thermal response also depends on both temperature magnitude and exposure duration [2, 18, 19, 20]. In living tissue, perfusion, vascular heat exchange, and post‐treatment biologic repair further modify the consequences of a given temperature exposure. Accordingly, although the observed ex vivo temperature magnitudes indicate substantial focal heat accumulation under the tested settings, they do not establish clinically meaningful injury thresholds or safe therapeutic windows.

Taken together, the present findings indicate that stacked multi‐wavelength diode irradiation can generate substantial subcutaneous and fascia‐level temperature elevations under non‐perfused experimental conditions. Rather than supporting prescriptive clinical parameter selection, these data provide an ex vivo reference for the relative magnitude and timing of local heat accumulation across different anatomical regions.

### 4.3 Interpretive Relevance for Body Contouring Energy Delivery

These findings may help inform how region‐specific energy delivery is interpreted in experimental and translational settings. In clinical practice, treatment parameters are often adjusted across body regions; however, such adjustments are commonly guided by empirical experience or general anatomical considerations rather than by direct measurement of deep‐plane heating. The present data show that both the timing and magnitude of fascia‐level heating can vary by anatomical site even under standardized irradiation conditions. This observation supports caution when assuming that identical settings will produce equivalent deep‐plane responses across different body regions.

From an ex vivo experimental perspective, the data also suggest that surface‐based indicators alone may not fully reflect deep‐plane thermal behavior. In regions with thicker adipose layers, delayed fascial heating may occur despite lower thermographic peaks in the exposed field, whereas in regions with thinner adipose layers, fascial heating may occur more rapidly under the same irradiation conditions. However, because cadaver tissue lacks perfusion and physiologic thermoregulation, these observations should not be interpreted as direct evidence of in vivo safety, tolerability, or cumulative thermal load. This limitation is particularly relevant from a bioheat transfer perspective, because in vivo temperature behavior is influenced not only by tissue conduction but also by perfusion‐mediated heat exchange, as described in Pennes‐type bioheat models. Accordingly, the present findings should be understood as representing non‐perfused ex vivo heat behavior rather than direct approximations of living tissue.

### 4.4 Limitations and Future Directions

This study has several important limitations. First, its cadaveric design precludes direct extrapolation to in vivo conditions because tissue perfusion and active thermoregulation, both of which critically influence heat dissipation and redistribution, are absent. Second, the sample size was extremely small, with only three cadavers included. For this reason, the statistical analyses were exploratory and should not be interpreted as robust inferential evidence. Third, temperature was measured at a single deep reference plane only. Although this approach allowed assessment of fascial arrival time and cumulative temperature behavior at depth, it did not permit characterization of temperature gradients across the dermis, fat, and intermediate tissue layers. Fourth, no histologic assessment was performed; accordingly, the study does not confirm adipocyte injury, protein denaturation, or other biologically meaningful tissue effects at depth. Fifth, no validated maximum depth of thermal effect was established, and the study does not support a direct claim regarding treatment depth beyond the measured fascial plane. Sixth, the use of fresh‐frozen cadavers, although advantageous for preserving gross tissue architecture, may still alter tissue thermal conductivity relative to living tissue. Finally, no formal heat‐transfer simulation or Arrhenius‐based thermal damage modeling was performed. Therefore, although the present data provide direct measurements at the fascial plane, they do not define injury thresholds or the full spatial pattern of heat distribution across intervening layers.

Future studies incorporating larger specimen numbers, multi‐depth temperature acquisition, in vivo monitoring, and region‐specific protocol modulation are warranted. Studies combining temperature measurement with histologic evaluation would be particularly valuable for defining thermal gradients across tissue layers and clarifying the biologic significance of measured heat exposure. In addition, finite element heat‐transfer simulation and thermal damage modeling may help further characterize regional heat distribution and biologically meaningful thermal thresholds during multi‐wavelength diode laser delivery. Integration of fascia‐level kinetic measurements with clinical outcome data will be required before any protocol recommendations can be made.

## 5 Conclusion

This cadaveric study demonstrates that fascia‐level thermal behavior during multi‐wavelength diode laser irradiation varies by anatomical region under non‐perfused ex vivo conditions. The anterior thigh showed the fastest deep fascial arrival, followed by the lateral upper arm, whereas the abdomen demonstrated the slowest fascia‐level heating under the tested settings. Greater subcutaneous fat thickness was associated with slower fascial heat arrival, but did not fully explain the observed regional variation. These findings should be interpreted descriptively: the study does not establish volumetric heat distribution, tissue injury thresholds, or clinical treatment optimization. Rather, it provides an ex vivo reference for region‐specific differences in fascia‐level heat arrival and cumulative temperature behavior that warrant further investigation in larger and in vivo studies.

## Author Contributions

All authors have reviewed and approved the article for submission. Kyu‐Ho Yi and Yerin Park conceptualized the study. Kyu‐Ho Yi and Yerin Park developed the methodology. Yerin Park, Hugues Cartier, Sebastien Garson, and Benjamin Ascher performed the investigation and acquired data. Yerin Park curated and analyzed the data. Yerin Park drafted the manuscript. Kyu‐Ho Yi, Hugues Cartier, and Sebastien Garson contributed to critical manuscript review and editing. Yerin Park prepared the visualizations. Kyu‐Ho Yi supervised the project and approved the final version.

## Funding

The authors have nothing to report.

## Ethics Statement

This study was approved by the Public Institutional Review Board designated by the Ministry of Health and Welfare, Republic of Korea (IRB No. P01‐202511‐01‐062) and was conducted in accordance with the Declaration of Helsinki. All cadaveric specimens were obtained from the body donation program of the Department of Anatomy and Developmental Biology, Yonsei University College of Dentistry (Seoul, Republic of Korea) and were handled in accordance with institutional policies and applicable regulations. Written informed consent for publication of clinical images was obtained from the participant. This study was conducted and reported in accordance with the STROBE guidelines.

## Consent

The authors have nothing to report.

## Conflicts of Interest

The authors declare no conflicts of interest.

## Data Availability

The data that support the findings of this study are available on request from the corresponding author. The data are not publicly available due to privacy or ethical restrictions.
